# Role of Microglia in Herpesvirus-Related Neuroinflammation and Neurodegeneration

**DOI:** 10.3390/pathogens11070809

**Published:** 2022-07-19

**Authors:** Magdalena Patrycy, Marcin Chodkowski, Malgorzata Krzyzowska

**Affiliations:** Military Institute of Hygiene and Epidemiology, 01-163 Warsaw, Poland; magdalena.patrycy@wihe.pl (M.P.); marcin.chodkowski@wihe.pl (M.C.)

**Keywords:** neuroinflammation, HSV, Herpesviridae, microglia, apoptosis, neurodegeneration

## Abstract

Neuroinflammation is defined as an inflammatory state within the central nervous system (CNS). Microglia conprise the resident tissue macrophages of the neuronal tissue. Upon viral infection of the CNS, microglia become activated and start to produce inflammatory mediators important for clearance of the virus, but an excessive neuroinflammation can harm nearby neuronal cells. Herpesviruses express several molecular mechanisms, which can modulate apoptosis of infected neurons, astrocytes and microglia but also divert immune response initiated by the infected cells. In this review we also describe the link between virus-related neuroinflammation, and development of neurodegenerative diseases.

## 1. Introduction

In contrast to other tissues, immune response within the central nervous system (CNS) is tightly controlled by different and unique immunomodulatory mechanisms: the presence of the blood–brain barrier (BBB), consisting of endothelial cells interacting with the glia limitans lining the CNS parenchyma, the lack of a typical lymphatic drainage system despite the presence of functional lymphatic vessels, as well as the lack of constitutive MHC expression [1,2]. Under physiological conditions, immune responses within the CNS are suppressed to protect neurons, which are vulnerable and nonrenewable cells. When a pathogen enters the CNS, resident and innate immune cells become activated to develop innate immune responses, which further result in the recruitment of adaptive immune cells from the periphery. However, the sustained and overactive inflammatory response may result in neuropathological events [3,4].

## 2. Herpetic Infections of CNS

Herpesviridae is a family of double-stranded DNA viruses, of which seven are known to infect humans and may be the cause of neurological diseases: (i) herpes simplex virus 1 (HSV-1), (ii) herpes simplex virus 2 (HSV-2), (iii) varicella zoster virus (VZV), (iv) human cytomegalovirus (CMV), (v) Epstein-Barr virus (EBV), (vi) human herpesvirus 6 (HHV-6), and (vii) human herpesvirus 7 (HHV-7). Following infection, herpesviruses establish latent infection within specific tissues/organs, characteristic for each virus, and can potentially reactivate in the case of immunity suppression [5].

HSV-1 belongs to α-herpesviruses and causes painful or itchy sores and blisters (oral herpes). Approximately 60% to 95% of adults worldwide are infected. HSV-1 is associated mainly with infections of the orofacial area and the CNS. Infection is either lytic or latent. Infection of epithelial cells (skin and mucosa) leads to lytic replication with production of infectious virus, while during latency limited gene expression is detected (only LATs transcripts are produced) and there is no production of viral particles. Primary infection is accompanied by inflammation and tissue damage, causing the characteristic herpes blisters. HSV-1 mainly infects sensory neurons close to the site of primary infection, subsequently traveling retrogradely along the axon to the cell body in the trigeminal ganglia (TG), where it establishes latency [6]. Latent HSV-1 genomes are found in episomal forms within the nucleus, where HSV-1 DNA is chromatinized with heterochromatic histone marks, and only a small subset of viral genes is expressed [7]. During latency, latency-associated transcripts (LATs), a primary 8.3/9 kb transcript and two stable introns derived from rapid splicing of primary LAT are expressed. Additionally, latent virus produces several microRNAs (miRNA) that act synergistically with LATs to repress viral replication [8,9].

Because trigeminal neurons are pseudounipolar, new viral particles can also reach the CNS via anterograde transport. Specifically, one of the two branches of trigeminal ganglia neurons projects to the trigeminal nuclei in the brainstem. Next, the neuronal projections reach the thalamus and, from there, the sensory cortex. TG neurons have therefore been proposed as a direct route for HSV-1 entry to the CNS [6,7].

Herpes simplex encephalitis (HSE) is one of the most common forms of viral encephalitis, with mortality of 70% in untreated patients and up to 30% mortality in patients treated with antivirals, and is followed by a high incidence of neurological complications. Primary HSV-1 infection and HSV-1 reactivation account for one-third and two-thirds of all HSE cases, respectively. Many studies have supported the concept that during events such as immunosuppression, peripheral infection and stress, the virus repeatedly reactivates in TG/brain [6]. However, a growing body of evidence indicates that in humans milder/asymptomatic infections also occur, followed by latency and such repeated “mild” HSV-1 brain infections are linked with chronic neuroinflammation resulting in neuronal damage similar to that found in neurodegenerative disorders such as Alzheimer’s disease (AD) [7]. HSV-1 reactivation from latency usually is induced by multiple stimuli, such as physical or emotional stress, immunosuppression, brain traumas or exposure to UV light [7].

Genital HSV-2 infection is more common in women (approximately one out of five women 14 to 49 years of age) than in men (about one out of nine men 14 to 49 years of age) [10,11]. After establishing latency in the sacral ganglia and infection of the spinal cord, HSV-2 can reactivate, leading to virus shedding in the genital tract, which is normally limited by activation of the HSV-specific T-cell-memory and production of IFN-γ [11]. Recurrences of genital herpes can be asymptomatic. Nevertheless, the virus shedding within the genital mucosa in women can be detected [11]. HSV-2-caused encephalitis is predominant in immunocompromised patients, causing less than 10% of the HSE cases.

Neonatal HSV, defined as infection in a newborn within 28 days of birth, results from intrauterine, perinatal, or postnatal transmission of the virus; most cases are acquired perinatally. HSV-2 infection during pregnancy poses a significant risk to the developing fetus and newborn [12]. Neonatal HSV has only 40% survival rate and results in permanent sequelae (developmental delay, epilepsy, blindness and cognitive disabilities). HSV-2 causes higher morbidity in infants than HSV-1 [12].

Varicella zoster virus (VZV, HHV-3), is an α-herpesvirus infecting >90% of people worldwide. It causes varicella (chickenpox) in unvaccinated children and establishes a lifelong latent infection in trigeminal ganglia (TG) and dorsal root ganglia (DRG) [13]. After many years, VZV may reactivate to cause herpes zoster (shingles) in approximately one third of previously infected individuals [14]. Encephalitis occurs in less than 0.1% of varicella cases, being thus a rare complication [15].

Epstein-Barr virus (EBV, HHV-4) is a γ1 human herpesvirus (HHV-4) that causes an infectious mononucleosis (IM), usually in children or adolescents. It affects up to 95 percent of humans worldwide, later transforming into chronic active EBV infection. EBV usually resides in a small number of circulating resting memory B-cells without causing any clinical symptoms (it establishes latency) [16,17].

Human CMV (HCMV, HHV-5) is a β-herpesvirus, which may cause a life-threatening infection in the immunocompromised, such as HIV-infected persons, organ transplant recipients, or newborn infants [18]. Studies on CMV-affected brain tissues showed that the basal ganglia, diencephalon and brainstem are the sites where CMV can be detected [19].

Human herpesvirus 6 (HHV-6) is a β-herpesvirus with two biologically distinct variants (HHV-6A and HHV-6B), showing different tropisms. HHV-6A has been described as more neurovirulent and it has been strongly associated with pathogenesis of multiple sclerosis [20,21]. HHV-6B causes a common childhood disease—exanthema subitum (also known as roseola infantum) [22,23].

Human herpesvirus-7 (HHV-7) is a β-herpesvirus, which might elicit exanthem subitem, although less frequently than HHV-6B [24,25]. HHV-7 can infect the brain and occasionally induce febrile convulsions or encephalitis [24,25].

## 3. Neuroinflammation

Inflammation is an innate response of the immune system in reaction to tissue injury or infection, which should result in pathogen elimination and/or tissue repair [26]. Innate immune cells, including monocytes/macrophages, mast cells, dendritic cells and microglia detect molecular patterns of microbes (PAMPs) and/or molecules from damaged cells of host origin (DAMPs) using pattern recognition receptors (PRRs). Activation of PRRs leads to activation of innate immune cells and production of reactive oxygen species (ROS) and reactive nitrogen species (RNS) responsible for killing microbes, release of stimulatory (pro-inflammatory) cytokines, chemokines and growth factors as well as for phagocytosis of cellular and tissue debris [27].

Despite “the immune privilege of the brain,” neuroinflammation takes the form of inflammation-like responses within the CNS, resulting in sustained production of cytokines, chemokines, ROS and RNS [2,3]. It is based on functional activation of microglia and astrocytes (the latter being the most abundant type of neural cells) [2,3], but also on infiltration of peripheral immune cells from the circulation: inflammatory monocytes—macrophages and antigen-specific T-lymphocytes resulting from the permeabilization of the BBB [2,3,28].

The glia consist of astrocytes (of neuronal origin) and microglia (immune cells); these two types differ in their activity and the role they play in the homeostasis of CNS. Microglia originate from yolk sac-derived macrophage progenitors that migrate to the brain at the early stages of embryonic development before the blood–brain barrier is fully formed, and comprise approximately 5–12% of the total cell population in the mammalian brain [29,30]. Microglia are crucial in maintaining brain homeostasis [29], but these cells also participate in the protection of the brain parenchyma from invading pathogens, utilizing different mechanisms, such as phagocytosis, the release of cytokines, chemokines, and antigen presentation via the major histocompatibility complex class II [29].

Microglia are a long-lived cell population involved in the refinement of synaptic connectivity by engulfing pre and postsynaptic structures [31]. Similarly to monocytes and macrophages in the periphery, microglia cells are defined as polarized, with two major activation states: pro-inflammatory M1 and anti-inflammatory M2 [32]. The M1 pro-inflammatory microglia are activated by brain-invading pathogens to produce pro-inflammatory cytokines such as: TNF-α, interleukin-1 beta (IL-1β), interleukin-6 (IL-6), interleukin-2 (IL-12), nitric oxide (NO), ROS, RNS, and superoxide [32]. The M2 microglia phenotype is induced by anti-inflammatory cytokines (IL-4 and IL-13) and it plays a crucial role in suppression of inflammation, phagocytosis of cell debris, toxic metabolites, and tissue repair [33]. M2 microglia produce anti-inflammatory IL-10, transforming growth factor (TGF)-β)], growth factors [insulin-like growth factor-1 (IGF-1), fibroblast growth factor (FGF) and colony stimulating factor (CSF)−1] [33]. However, M1 and M2 represent a spectrum of activation patterns rather than separate cell subtypes, since M1 can be converted into M2 by many modulators [33], and the lack of conversion of M1 to M2 is found in most CNS diseases [33]. Interestingly, LPS-activated microglia secreting IL-1α, TNF-α and C1q can induce a population of reactive astrocytes named A1. These astrocytes do not promote neuronal survival but rather lead neurons and oligodendrocytes to death. This phenomenon indicates that both types of glial cells can modulate their mutual activity [34].

## 4. Microglia in Viral Encephalitis

The activation of microglia and the infiltration of immune cells from the peripheral circulation into the HSV-infected CNS is crucial for limiting viral infection and infection-related inflammation. However, if the virus is not controlled early after infection, an uncontrolled immune response can develop and lead to high mortality and morbidity [35,36].

In recent years, the clinical genetic study of patients and their families with high incidence of HSE has demonstrated that single-gene errors of innate or cell-intrinsic immunity are responsible for enhanced susceptibility to HSV infections. The detected genetic defects showed that HSE in children results from reduced production of the IFN type I [37], both upon HSV-1 infection or ex vivo stimulation of the TLR3 pathway, in fibroblasts and specific CNS cell lineages derived from pluripotent cells [37]. During infection with DNA viruses like HSV, endosomal TLRs initiate IFN I signaling upon recognition of viral nucleotides (TLR3 recognizes double-stranded RNA, TLR8/7 recognizes single-stranded RNA, and TLR9 recognizes unmethylated cytosine-phosphate-guanine, or CpG, DNA). Additionally, cytosolic viral DNA is sensed by the cyclic GMP-AMP synthase/stimulator or interferon genes (cGAS/STING) pathway.

Mouse models lacking specific factors involved in IFN I signaling have helped to understand the role of TLR and cGAS/STING viral nucleotide sensing in the pathogenesis of HSE. After interacting with activated microglia in glial cultures, murine astrocytes can upregulate TLR2, TLR3, and TLR4, while microglia express TLRs both at steady-state and upon activation [38,39]. Intravaginal infection of Tlr3−/− mice with HSV-2 showed their higher susceptibility to HSE in comparison to wild-type (WT) mice [38]. On the other hand, upon HSV-1 infection, Tlr2−/− mice are resistant to HSE, and Tlr9−/− or double Trl2−/−Tlr9−/− knockout mice are partially and fully susceptible, respectively [40]. Therefore, TLR9 does not play a major role in the fight against HSV-1, but rather functions simultaneously with TLR2 leading to recruitment of natural killer (NK) cells to the sites of HSV infection in the CNS [41]. Since Tlr2 and Tlr9 gene expression is mostly upregulated in the trigeminal ganglia after HSV-1 infection, trigeminal ganglia play the role of a crucial checkpoint for viral recognition and control [42]. When using intracranial infection with HSV-1 of Tlr2−/−, Tlr9−/−, Trl2−/−Tlr9−/− mice to bypass the TG, researchers found that only Tlr2−/− mice were more resistant to HSV than WT mice [41].

Recent studies in knock-out mice have determined an important role for cytosolic viral DNA sensing in innate immunity to HSE. Several DNA sensors have been identified, including cyclic GMP–AMP synthase (cGAS) [43], and all type I IFN-inducing cytoplasmic DNA sensors signal through the adaptor protein stimulator of type I IFN genes (STING) [44]. Mice lacking either cGAS or STING are highly susceptible to HSE, and are characterized by high viral titers in the TG, brainstem, and further involvement of the whole brain [44]. Reinert et al. (2016) have shown that during HSV-1 infection of CNS, microglia are the main source of type I IFN, and transduce this response in a STING-dependent manner, irrespectively of productive infection [43].

Upon HSV-1 infection, microglial cells undergo an abortive infection followed by a burst of pro-inflammatory cytokine and chemokine production, which activate other glia cells within the CNS as well as attract immune cells, such as DCs, NK cells, and T cells, to the brain [2,3]. In other words, microglia play an important role in mounting of the adaptive immune response in the CNS.

In mice models of HSV-1 infection the number of microglia is increasing early during brain infection. The study by Uyar et al. showed that transient and incomplete depletion (6 day post infection) of microglial response decreased infiltration of monocytes/macrophages, neutrophils and T cells into the brain [45]. Similar results were obtained by Tsai et al. in a study where microglia were depleted by the use of PLX 5622, an inhibitor of colony-stimulating factor 1 receptor (CSF1R), necessary for microglia survival, growth, and proliferation [46].

Depletion of microglia before and during infection augments brain viral titers by about 10- and 100-fold on days 6–7 p.i. and increases mortality rates of infected mice by 60 and 85%, respectively [46]. Lack of microglia activation is accompanied by increased infiltration of CD4 T cells, CD8 T cells, neutrophils, and increased production of IFN-β, and IFN-γ [45,46]. However, these compensatory mechanisms cannot reduce brain viral loads and mice lethality [45,46]. The study by Tsai et al. showed in vitro that brain microglia induce the IFN-β-STAT1 signaling pathway, which suppresses viral replication and apoptosis of brain neurons [46].

**Figure 1:**
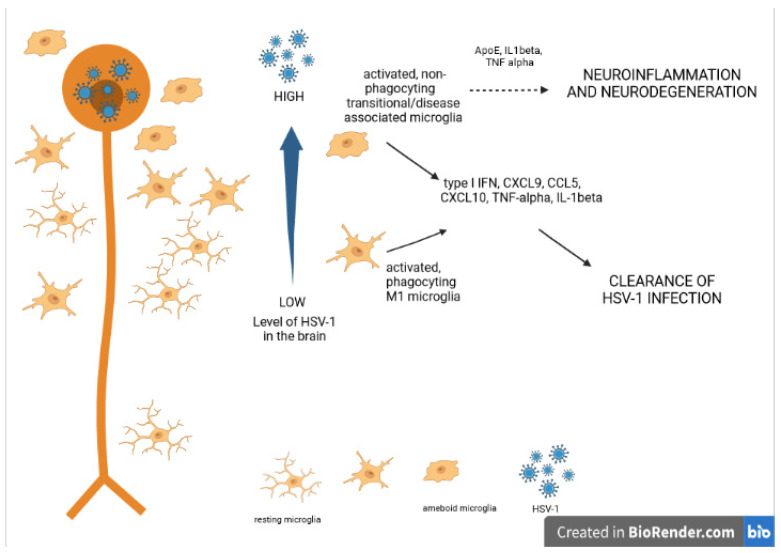
Schematic diagram summarizing relationships between HSV-1 brain infection, microglia activation and development of neuroinflammation and neurodegeneration. Accessed on 15 July 2022.

Another study by Uyar et al. demonstrated that, depending on the infection levels, microglia are highly phagocytic and participate in antigen presentation (moderately infected thalamic areas) or display impaired physiological functions, such as decreased phagocytic activity (highly infected thalamic regions) [47]. By using single-cell RNA sequencing (scRNA-seq) on cells isolated from highly infected thalamic regions of HSV-1 infected C57BL/6 mice, Uyar et al. revealed a novel transcriptional signature of microglia/microglia-like cells, which they defined as “in transition”. Pathway analysis of this cell-type transcriptome showed that “in transition” microglia/microglia-like cells detect the dsRNA of HSV-1 via RIG-I (retinoic acid-inducible gene I, also known as DDX58)/MDA5 in the cytosol), which results in type I IFN production and NLRP3 inflammasome-mediated IL-1β production [47] (Figure 1).

Another study by Fekete et al. demonstrated a rapid recruitment (within hours) and elimination of HSV-1 infected neurons via microglial P2Y12 receptors in vivo [48]. However, recruitment of leukocytes into the HSV-1-infected brain was independent of P2Y12-mediated signaling [48].

Moreover, Marques et al. showed that microglia remained activated in the brain of HSV1-infected mice at 30 days post infection, while no active replication could be detected [3]. The authors suggested that persistent microglia activation may contribute to neuronal damage and long-term neurological sequelae observed in HSE patients [3]. Multiple HSV1 reactivations in murine models of HSE are accompanied by gliosis, increased brain levels of IL-1b and IL-6 as well as increased expression of neurodegenerative markers (e.g., beta-amyloid, phosphorylated tau proteins) [49] (Figure 1).

Virus-activated microglia produce high amounts of CCL5, CXCL10, TNF, and IL-1β as well as lower amounts of IL-6, IL-8, CCL3, CCL4, and CCL2 in a TLR3-dependent manner [2,50]. Chemokines CCL5, CCL2, and CXCL10 further recruit peripheral immune cells to the infected brain [2,3,36], of which CXCL10 in particular has been shown to be important in mounting of HSV-1 specific T cell response [50]. IFN-β produced by microglia mediates the production of anti-inflammatory IL-10, which can suppress severe inflammation [35], while IL-6 is protecting from the loss of neurons [51].

As mentioned above, cytokines and chemokines produced by microglia may also show neurotoxic activity, as it has been demonstrated for TNF and IL-1β [50]. HSV-1-infected microglial cells produce ROS and RNS, which are directly responsible for damage to neurons [29]. Interestingly, while NO produced by microglial cells can help to reduce viral replication in HSV-1-infected neurons but not in astrocytes, it also down-regulates cytokines important for induction of anti-viral response in the neuronal tissue [52]. Therefore, sustained and increased local production of NO may further add to HSV-1 spread, and escalate further neuroinflammation by suppression of the specific anti-viral immune response. Chronic HSV-1 infection has been shown to facilitate persistent activation of microglia and neuronal damage [53].

In other types of neuronal infections by herpes viruses, microglia play a similar role. Studies using a mouse model of murine cytomegalovirus (MCMV) have demonstrated that following i.p. infection, the virus reaches the CNS via blood, replicates in the brain parenchyma, distorts cerebellar development, and induces a strong inflammatory response of microglia and infiltration of innate immune cells [53]. Cytomegalovirus-activated microglia have been shown to suppress MCMV replication in astrocytes by secreting IFN-α and TNF-α, but also by attracting NK cells and T cells in a CXCL9/CXCL10-dependent manner [53,54,55].

Increasing evidence points to an MS link, with detection of EBV in brains of MS patients [56]. For example, increased antibody response to an EBV-encoded EBNA1 protein is detected in developing MS compared to baseline IgG titers to EBNA1 [57]. Furthermore, almost all MS patients are infected with EBV compared to ~95% non-MS controls [57].

One of the concepts supporting the role of EBV in pathogenesis of MS is that latent EBV infection leads to neuroinflammation by inducing IFN-α production. This is supported by the data showing that EBERs (EBV encoded small RNAs) can bind to TLR3 and other intracellular receptors such as retinoic acid-inducible gene 1 (RIG-I) and promote production of IFN-α [58]. The detection of EBER+ cells in active MS within the white matter lesions was linked with strong expression of IFN-α by cells showing the ameboid morphology of microglia and macrophages [57]. Microglia and astrocytes activated in MS lesions, however, do not seem to be directly infected with EBV [17].

In contrast, Hassani et al. detected presence of EBV in brain tissues from MS and non-MS-cases using PCR and EBER-in situ hybridization and demonstrated that EBV could be detected by PCR and/or EBER-ISH in 90% of MS cases compared to 24% of non-MS cases with other neuropathologies. No other common herpesviruses (HSV-1, CMV, HHV-6) were detected [59]. Additionally, in contrast to previous studies, Hassani et al. found astrocytes and microglia infected with EBV. The route of microglia and astrocyte infection by EBV is unknown and needs to be elucidated [59].

HHV-6A can productively infect microglial cells both in monolayer and spheroids; it induces activation as shown by the increased expression of triggering receptor expressed in myeloid cells 2 (TREM2) and IL-1beta [60]. The further link between HHV-6 induced microglia activation and neuroinflammation needs further study.

## 5. Cell Death and Neuroinflammation

Neuronal apoptosis has been also suggested to contribute to the CNS injury in HSE. Apoptosis is an active, genetically controlled process, which can be triggered by a variety of extrinsic and intrinsic signals such as toxins, inflammatory cytokines, lack of growth factors, ROS, and infectious pathogens. Morphologically, apoptosis leads to cellular shrinkage and chromatin condensation that result in the controlled breakdown of the cell into apoptotic bodies, which are subsequently recognized and engulfed by surrounding cells and phagocytes [61]. Two major signaling pathways have been discovered: receptor-ligand mediated pathway (Fas/FasL pathway) and intracellular mitochondrial pathway. These pathways are regulated by abundant pro- and anti-apoptotic proteins and compounds [61].

**Figure 2:**
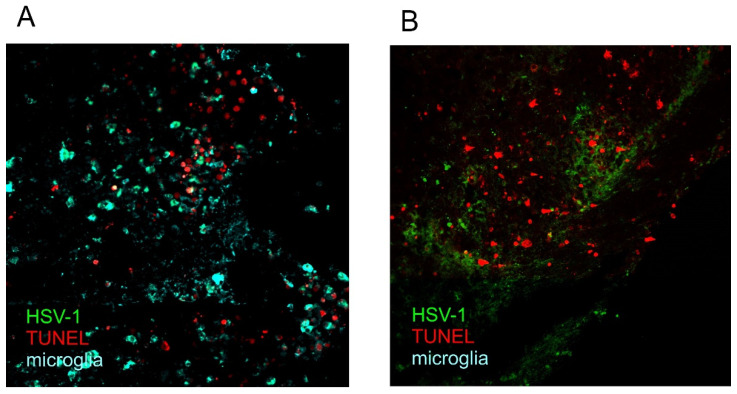
Presence of HSV-1-positive and apoptotic (TUNEL+) cells in the midbrain (**A**) and pons (**B**) of C57BL/6 mice at 8 days post infection (unpublished data from authors). C57BL6 mice were infected intranasally with McKrae strain of HSV-1, then followed until the peak of brain infection at day 8. Next, brains were subjected to cryopreservation, sectioning and immunofluorescent staining for IBA+ positive cells (microglia, turquoise), HSV-1 antigens (green) and terminal deoxynucleotidyl transferase dUTP nick end labeling (TUNEL) to detect apoptotic DNA fragmentation (red). Magnification × 200.

During acute brain infection in HSE, HSV-1 was demonstrated to induce apoptosis in neural cells, which was believed to contribute to virus induced brain damage [62]. In analyzed brains of patients with HSE, the executory phase of apoptosis was detected by the TUNEL method (terminal deoxynucleotidyl transferase-mediated dUTP nick-end labeling) [62]. Furthermore, apoptosis was detected by TUNEL staining in the brainstems of HSV-1 infected mice, where a significant amount of apoptotic staining was observed at day 6 post-infection, despite the low levels of detectable infectious virus [63]. Studies performed in C57BL6 mice infected intranasally with a clinical isolate of HSV-1 by our group also showed the presence of apoptotic, TUNEL-positive cells related with inflammatory reaction (Figure 2A). However, no apoptosis was detected in inflammation-free areas with HSV-1-infected neurons (Figure 2B).

HSV-1 has been shown to encode several proteins, which can modulate—induce or suppress—apoptosis at different stages of viral replication, depending on the cell type. The structural glycoproteins J and D (gJ, gD) have been reported to suppress apoptosis during HSV-1 infection of the SK-N-SH human neuronal cell line [64]. ICP22 is another HSV-1 protein involved in modulation of apoptosis—it induces cell death by activating Bax or inhibiting Bcl-2 [65]. The viral ICP27 has been reported to block caspase 3 activity during the executory phase of apoptosis [66]. When HSV-1 establishes latency in neurons, it expresses LAT transcript, and the miRNAs derived from the LAT processing have also been demonstrated to modulate apoptosis in infected neurons. LAT expressed in a plasmid has been shown to inhibit apoptosis induced via receptor and mitochondrial pathways [67]. These observations are related to the observed in vivo inhibition of CD8+ T cell-killing of latently infected neurons, as LAT expression could block granzyme B-induced activation of caspase 3 [67]. Inhibition of apoptosis seems to play a key role in neurodegeneration processes induced by HSV-1, since it favors the establishment of latency and persistence with later reactivations and spread into the neighboring neuronal tissue, leading to neuroinflammation-induced neuronal damage.

Reinert et al. (2021) showed extensive apoptosis in HSV-1–infected brains originating from HSE patients and in the experimental HSE mouse model [68]. Apoptosis induction was observed in microglia and other immune cells and the cell death was mediated by the DNA-sensing cGAS/STING pathway. The use of caspase inhibitors increased cGAS-dependent antiviral activity in the brain and was also accompanied by prolonged and increased production of IFN-I by immune cells [68].

The Fas (CD95, APO-1)-signaling pathway is a so called receptor apoptotic pathway. Fas and FasL play critical roles in the immune system, being the direct mechanism responsible for the killing of pathogen-infected target cells and the death of autoreactive lymphocytes [69]. Fas is not normally expressed on central nervous system (CNS) cells; however, its expression can be induced within inflammatory sites, resulting in their susceptibility to FasL-induced death [69,70]. Within the CNS, FasL expression is detectable in neurons, microglia and perivascular astrocytes [71].

Upon stimulation of HSV-1 infected microglia through Fas, they become resistant to Fas-mediated apoptosis and down-regulate inflammatory response—production of pro-inflammatory and anti-viral cytokines and chemokines such as CCL2, CXCL9, CXCL10, IFN-α, TNF-α and IL-6 [36]. HSV-1 infected in vitro cultures of microglia isolated from Fas- and FasL-deficient mice show strong inflammatory responses in comparison to microglia derived from wild type mice [36]. Therefore, we propose that HSV-1 can interfere with Fas-mediated pro-inflammatory pathways within the CNS leading to disturbances in Fas-mediated apoptosis and pro-inflammatory response upon migration of FasL bearing lymphocytes into the infected neuronal site [36].

Astrocytes undergo productive HSV-1 replication, but did not undergo immediate cell death. Recently, Jeffries et al. (2022) showed that murine astrocytes express Z-DNA binding protein 1 (ZBP1; also known as DNA-dependent activator of interferon regulatory factors (DAI)) [72]. The loss of ZBP1 expression in primary murine astrocytes results in the significantly increased release of virions upon infection with a neuroinvasive clinical strain of HSV-1 [72]. The authors also demonstrated that inhibition of apoptotic pathways leads to HSV-induced necroptosis in astrocytes that is independent of ZBP1 [72]. Both necroptotic and apoptotic cell death pathways in HSV-1 infected astrocytes reduce release of infectious viral particles [72].

## 6. Neuroinflammation and Neurodegeneration

Several studies have provided evidence of associations between infections with herpetic viruses, a decline in cognitive abilities, and Alzheimers disease (AD). AD is an inflammatory brain disease associated with a combination of environmental agents and genetic predispositions leading to neuroinflammation, neuronal cell death, and progressive dementia. The vast majority of AD occurs on a sporadic basis, while certain mutations cause a rare (<0.5%) familial form of AD [73,74]. The main features of Alzheimers pathology are amyloid plaques made up of amyloid-β (Aβ), a peptide cleaved from APP precursor, and intracellular neurofibrillary tangles (NFTs) consisting of abnormally phosphorylated tau protein [74]. Furthermore, neutrophil infiltration, dystrophic neurites, associated astrogliosis and microglial activation are observed [74]. Aβ is toxic to neurons as it may cause pore formation resulting in disruption of cellular calcium balance, and loss of membrane potential. It promotes apoptosis, causes synaptic loss, and disrupts the cytoskeleton. Accumulation of hyperphosphorylated tau protein impairs synaptic function, induces mitochondrial stress and activates microglia [75].

The AD pathogen hypothesis states that pathogens invading the CNS act as triggers, interacting with genetic factors to initiate neuroinflammation and accumulation of Aβ as well as of hyperphosphorylated tau proteins in the brain tissue [76]. Previous studies demonstrated the presence of HSV-1 DNA in the amyloid plaques found in the brains of AD patients, particularly of those carrying the ε4 allele of apolipoprotein E (APOEε4) [76]. Many population-based studies found a link between the level and avidity index of anti-HSV-1 IgG and IgM serum antibodies (indicating previous HSV-1 infection and its reactivation, respectively) and the risk of developing AD [77]. On the other hand, Carbone et al. analyzed DNA from peripheral blood leukocytes (PBL) and brain samples of AD patients for the presence of CMV, EBV, or HHV-6. All samples were negative for CMV, while EBV was detected in 6% of AD brains. HHV-6 showed a 23% positivity in blood leucocytes in up to 17% of AD patients’ brains, compared to 4% of controls [78]. Another study tested hundreds of brains and reported a greater abundance of HHV6 or HHV7 RNA and DNA in the brains of AD patients relative to controls [79].

**Figure 3:**
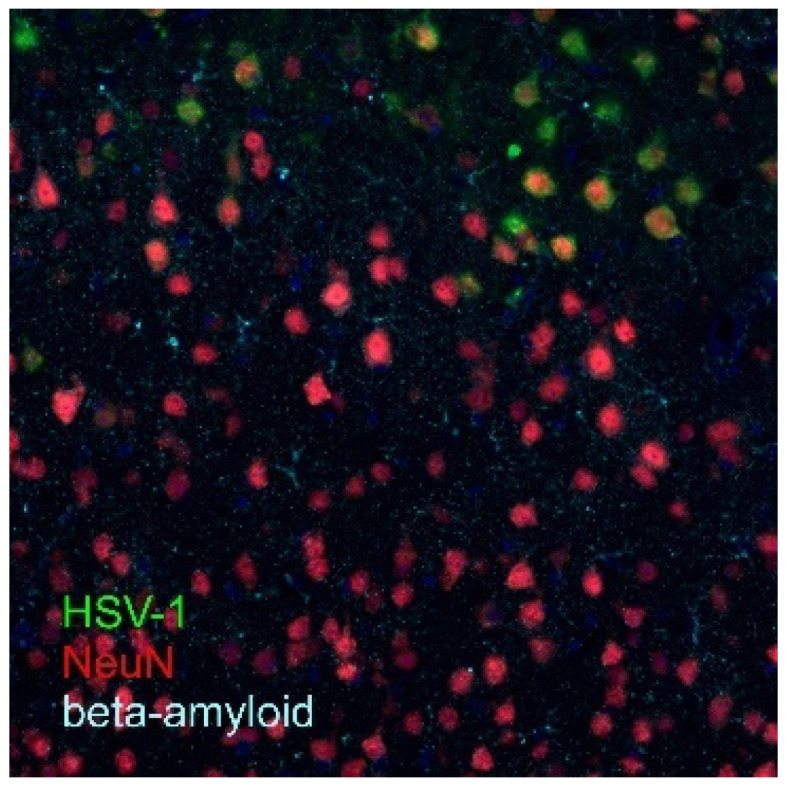
Presence of HSV-1 and β-amyloid in the midbrain of C57BL/6 mice at 8 days post infection (unpublished data from authors). C57BL6 mice were infected intranasally with McKrae strain of HSV-1, then followed until the peak of brain infection at day 8. Next, brains were subjected to cryopreservation, sectioning and immunofluorescent staining for neurons using anti-NeuN antibody (red), anti- HSV-1 antigens (green) and anti-1-42 β-amyloid antibody (turquoise). Magnification × 200.

More recent studies have focused on investigating a direct link between HSV-1 infection and AD pathogenesis both in vitro and in vivo. HSV-1 infection in cultured neurons induces processing of APP followed by intra- and extra-neuronal accumulation of Aβ and other neurotoxic APP fragments [80]. HSV-1 infected neuroblastoma cells also accumulate hyperphosphylated tau protein within the nucleus, a component of neurofibrillary tangles, another characteristic feature of AD [81]. Mouse brains infected with HSV-1 also show increased Aβ deposition early after infection (Figure 3).

HSV-1 infection of 5XFAD mice that overexpress mutant human APP with familial AD mutations did not show any alteration of the infection patterns [82]. Furthermore, 5XFAD mice that survived infection cleared HSV-1 virus in the brain areas susceptible to virus without triggering extracellular Aβ aggregation, mainly due to microglia activity [82]. However, in another study, HSV-1 was found to colocalize with Aβ plaques 3 weeks post infection in young 5XFAD mice that survived the challenge [83]. Authors of the first study suggest that the actual results may strongly depend on the overloading of a brain with very high, non-physiological doses of HSV-1 [82].

On the other hand, De Chiara et al. (2019) established a mouse model of HSV-1 infection and reactivation to verify if multiple viral reactivations, experienced by some patients prone to recurrent infections, can trigger progressive accumulation of molecular markers of neurodegeneration with an accompanying cognitive decline similar to this observed in Alzheimer’s disease [49]. Indeed, following virus reactivations, HSV-1 actively replicated in the brain, accompanied by progressive accumulation of AD biomarkers in neocortex and hippocampus of infected mice; and AD biomarker accumulation was associated with cognitive impairment. These biochemical and functional alterations increased with the number of virus reactivations [49].

In the presence of endogenous or exogenous insults to the neuronal tissue (mechanical trauma, infection, accumulation of toxic metabolites) ramified, “resting” microglia transform into amoeboid, “activated” microglia [83]. Ameboid microglia participate in the clearance of potentially dangerous pathological situations in the neuronal tissue by phagocytosis, endocytosis, and secretion of various inflammatory mediators [84] (Figure 1).

Neuroinflammation is now well recognized as an important pathological process leading to AD and a potential target for therapy and prevention. Microglia and astroglia are found surrounding amyloid plaques in AD brains [85] although microglia can play a dual role in Aβ pathogenesis. Microglia help eliminate Aβ aggregation via phagocytosis; on the other hand, amyloid deposition causes a microglial-mediated inflammatory response [85,86]. Although early microglial recruitment can promote Aβ clearance and prevent the pathologic progression in AD, a constant microglial accumulation is associated with the release cytotoxic molecules (such as proinflammatory cytokines, chemokines, ROS, RNS), which can in turn promote Aβ production and delay the Aβ clearance [85,86].

In transient states of pathologies such as ischemia, CD11-positive microglia change their phenotype to the pro-inflammatory M1 phenotype, followed by a switch to M2 phenotype with anti-inflammatory characteristics related with tissue-repair [87]. As mentioned above, and depending on in vivo pathology, M1 and M2 microglial phenotypes are mutually interconvertible [88].

The neurodegenerative diseases (like AD) as well as aging are associated with chronic neuroinflammation. For those conditions, a pro-inflammatory phenotype of microglia has been characterized and designated “disease-associated microglia” (DAM). This phenotype does not have the typical M1 phenotype (for example, ApoE is down-regulated in M1 but up-regulated in DAM), but it also demonstrates some similarity to the M2 phenotype (such as up-regulated Arg-1 expression found in both DAM and M2) [89] (Figure 3).

Taking into account the devastating character of AD, and the associated significant economic burden to the families and society, as well as the increasing numbers of older people, there is a constant need to develop new treatment strategies for AD. These strategies should focus on the regulation of neuroinflammation-related pathologies.

Parkinson’s disease (PD) is the second most frequent neurodegenerative disease, affecting more than 1% of individuals older than 65 years. The main pathological features of PD are degeneration of nigrostriatal dopaminergic neurons, reduction of striatal dopamine, and the formation of abnormal protein aggregates in neurons, such as Lewy vesicles. PD is a multifactorial disorder whose pathogenesis involves such factors as neuroinflammation, oxidative stress, infection, and genetic factors [90]. Epidemiological studies have demonstrated that patients with PD are significantly more seropositive for EBV than the general population. Latent EBV infection can trigger autoantibodies that can cross-react with α-synuclein and elevate α-synuclein aggregation [91]. LMP1 is a virally encoded membrane protein expressed during EBV latency, which shares a similar protein primary amino acid PXDPDN sequence with α-synuclein [91].

Despite much research on the pathogenesis and treatment of PD, the mechanisms of EBV involvement in PD development are poorly understood, and further research is necessary.

## 7. Conclusions

Evidence from epidemiological and experimental studies suggests the existence of a link between herpesvirus brain infection and neurodegenerative processes such as AD and MS. Most of these data were obtained in animal models, and further studies are required to validate this correlation in humans. Furthermore, little is known about how the virus- and host-related factors (viral yield, strain, genetic features, concurrent infections, diseases) contribute to virus spread to the brain, development of neuroinflammation and further neurodegeneration. Conflicting results were obtained by different research groups concerning the role of beta-amyloid in HSV-1 infection and the presence of HSV-1 in amyloid plaques. Similarly, there is still no clear explanation of the role and presence of other herpesviruses, such as HHV-6 and EBV, in neurodegenerative processes. Without answering the above questions, finding novel strategies to limit virus reactivation and diffusion to the brain may not bring rapid clinical solutions to stop neuroinflammation and related neurodegeneration.
